# Family history of breast and ovarian cancer and triple negative subtype in hispanic/latina women

**DOI:** 10.1186/2193-1801-3-727

**Published:** 2014-12-11

**Authors:** Kristin Anderson, Patricia A Thompson, Betsy C Wertheim, Lorena Martin, Ian K Komenaka, Melissa Bondy, Adrian Daneri-Navarro, Maria Mercedes Meza-Montenegro, Luis Enrique Gutierrez-Millan, Abenaa Brewster, Lisa Madlensky, Malaika Tobias, Loki Natarajan, María Elena Martínez

**Affiliations:** Moores Cancer Center, University of California San Diego, 3855 Health Sciences Dr., #0901, La Jolla, CA 92093-0901 USA; University of Arizona, Tucson, AZ USA; Department of Family and Preventive Medicine, University of California San Diego, La Jolla, CA USA; Maricopa Medical Center, Phoenix, AZ USA; Baylor College of Medicine, Houston, TX USA; Universidad of Guadalajara, Guadalajara, México; Instituto Tecnológico de Sonora, Ciudad Obregón, México; Universidad of Sonora, Hermosillo, México; University of Texas M.D. Anderson Cancer Center, Houston, TX USA

**Keywords:** Triple negative breast cancer, Hispanic, Family history, Risk factor heterogeneity

## Abstract

Familial breast and ovarian cancer prevalence was assessed among 1150 women of Mexican descent enrolled in a case-only, binational breast cancer study. Logistic regression was conducted to compare odds of triple negative breast cancer (TNBC) to non-TNBC according to family history of breast and breast or ovarian cancer among 914 of these women.

Prevalence of breast cancer family history in a first- and first- or second-degree relative was 13.1% and 24.1%, respectively; that for breast or ovarian cancer in a first-degree relative was 14.9%. After adjustment for age and country of residence, women with a first-degree relative with breast cancer were more likely to be diagnosed with TNBC than non-TNBC (OR=1.98; 95% CI, 1.26–3.11). The odds of TNBC compared to non-TNBC were 1.93 (95% CI, 1.26–2.97) for women with a first-degree relative with breast or ovarian cancer. There were non-significant stronger associations between family history and TNBC among women diagnosed at age <50 compared to ≥50 years for breast cancer in a first-degree relative (*P*-interaction = 0.14) and a first- or second-degree relative (*P*-interaction = 0.07).

Findings suggest that familial breast cancers are associated with triple negative subtype, possibly related to *BRCA* mutations in Hispanic/Latina women, which are strongly associated with TNBC. Family history is an important tool to identify Hispanic/Latina women who may be at increased risk of TNBC, and could benefit from prevention and early detection strategies.

**Electronic supplementary material:**

The online version of this article (doi:10.1186/2193-1801-3-727) contains supplementary material, which is available to authorized users.

## Background

Having a first-degree relative with breast cancer is associated with a 2-fold increase in risk of developing the disease, despite the fact that mutations in high-risk alleles such as *BRCA1/2* explain less than 20% of familial cancers (Collaborative Group on Hormonal Factors in Breast, C 2001, Mavaddat et al. 2010). Breast cancer is a heterogeneous disease with differences in patient outcomes based on tumor hormone receptor status and expression of human epidermal growth factor receptor 2 (HER2). Gene expression studies have confirmed the existence of at least four distinct and reproducible breast cancer subtypes with molecular differences based on these markers (Perou et al. 2000). These differences have resulted in stratification in the clinical setting with varying treatment algorithms based on subtype (Paik et al. 2006, Slamon et al. 2011).

Epidemiological studies provide additional evidence supporting differential effects of reproductive risk factors on risk of developing hormone receptor positive or negative tumors, as we recently reviewed (Anderson et al. 2014), which also include case-only studies (Martinez et al. 2010). Non-reproductive risk factors, such as obesity, have also been shown in a recent meta-analysis to confer a greater risk for triple negative breast cancer (TNBC) compared to non-TNBC, particularly among pre-menopausal women (Pierobon and Frankenfeld 2013). Results of some studies, which are reviewed here (Additional file 1: Table S1), have also shown differences in risk by subtype according to family history, but results are inconsistent. These findings underscore heterogeneity in etiology among breast tumors. Furthermore, genetic variation in susceptibility to breast cancer within certain racial/ethnic populations, along with interactions of these heritable risk factors with environmental and reproductive risk factors, may contribute to differences in breast cancer risk and outcomes by tumor subtype.

Relatively little data exist on risk of breast cancer associated with family history of breast and ovarian cancers in Hispanic/Latina women, and fewer reports have been published on associations by tumor subtype (Hines et al. 2008, Jiang et al. 2012). Furthermore, there is a paucity of information on the family history profile of breast and ovarian cancer in this ethnic group. Such information could help identify the degree of misreporting due to lack of awareness or limited family structure, resulting from few female family members who have survived to older ages (Weitzel et al. 2007a). Importance of improved family history ascertainment relates to the recent publication of Hispanic/Latina breast and ovarian cancer families in the U.S. that showed a high prevalence of *BRCA* mutations (25%) (Weitzel et al. 2013).

To address these important gaps, here we first describe the self-reported breast and ovarian family history profile in a breast cancer case series of women of Mexican descent. We then assess odds of TNBC according to family history of breast and ovarian cancer.

## Methods

### Study population

The *Ella* Binational Breast Cancer Study is a case-only study of invasive breast cancer; details of the study have been previously described (Martínez et al. 2010). Mexican and Mexican-American women age 18 years or older were recruited within 24 months of diagnosis. Recruitment sites included two in the U.S. (the University of Arizona Cancer Center in Tucson, Arizona and the M.D. Anderson Cancer Center in Houston, Texas) and three in Mexico (the Universidad de Sonora in Hermosillo, Sonora; the Instituto Tecnológico de Sonora in Ciudad Obregón, Sonora; and the Universidad de Guadalajara in Guadalajara, Jalisco). All sites used a predominately clinic-based recruitment strategy. Recruitment occurred from March 2007 through June 2011, with response rates ranging from 95-99% (Martínez et al. 2010). The Institutional Review Board from each participating institution approved the study protocol, and all women provided informed consent. IRB approvals: 1. UCSD Human Research Protections Program; 2. University of Arizona Human Subjects Protection Program; 3. The University of Texas MD Anderson Cancer Center Institutional Review Board; 4. Commission on Ethics, Investigation and Biosafety, University of Guadalajara; 5. Committee on Ethics, Instituto Jaliscience de Cancerologia; 6. National Commission on Scientific Research of the Instituto Nacional del Seguro Social; 7. Bioethics Institutional Committee, Instituto Tecnologico de Sonora.

### Data collection and variable definition

Risk factor data were ascertained from an interview-administered questionnaire that included sociodemographics, reproductive history, family history of cancer, and other risk factor data. Details were previously published (Martínez et al. 2010, Martinez et al. 2013) and are further described below.

Comprehensive family history data were collected to include first-degree (brother, sister, son, daughter, father, mother) and second-degree (grandfather, grandmother, uncle, aunt) relatives. Half-siblings were not included. A total of 1150 participants were asked, “Have any of your immediate family members ever been diagnosed with cancer?” Those who answered “yes” (*n* = 612) were then asked to report the type of cancer for each family member and his/her age at diagnosis. Women who answered “no” (*n* = 510) or “don’t know” (*n* = 28) did not supply any additional information regarding family history of cancer. We also asked participants whether they were adopted and, if so, whether they knew her blood relatives. A woman who reported that she was adopted and did not know her blood relatives was categorized as “don’t know”; adopted women who knew their blood relatives were categorized as positive or negative for family history according to their response.

For breast or ovarian cancer family history, we counted the number of women who reported a history of these malignancies in first- or second-degree relatives. Women who reported a female relative with cancer of unknown type were classified as “unknown” for the relevant family history variables, depending on whether the relative in question was first or second degree, which contributed to differences in the denominators available for each variable.

### Clinical data

Age at diagnosis and tumor marker data for estrogen receptor (ER), progesterone receptor (PR), and HER2 were abstracted from medical records. In the abstraction, priority was given to a numeric value for the percent of cells staining, where ER and PR positivity was defined as ≥ 1% cell staining by immunohistochemistry (IHC). Cases were considered HER2+ if amplified as determined by fluorescence *in situ* hybridization (FISH; ratio ≥ 2.2). If no FISH results were available, an IHC intensity score of 3/3+ was considered positive, 2/2+ equivocal, and 0/1/1+ negative. If the IHC intensity score was 3/3+, that tumor was classified as HER2+ regardless of FISH value. Tumors with an equivocal HER2 IHC intensity score and no FISH data were excluded (n = 53). Cases were categorized as either TNBC (ER–, PR–, and HER2–) or non-TNBC, which included luminal A (ER+ and/or PR+ and HER2–), luminal B (ER+ and/or PR+ and HER2+), and HER2+ (ER-/PR- and HER2+) cancers. Of the 1150 women who were asked about their family history, 960 had known TNBC status, of which 914 had known status for family history of breast cancer in a first- or second-degree relative.

### Statistical analysis

Descriptive statistics (mean ± SD and proportions) for family history and risk factor characteristics were calculated. Associations between family history and TNBC status were tested using logistic regression, using non-TNBC as the reference group. Since the primary objective was to quantify the associations between presence of family history of breast cancer and TNBC as a means of understanding tumor heterogeneity rather than building risk models, the models were adjusted only for age at diagnosis (continuous) and recruitment country (U.S. or Mexico). In addition, we found minimal differences in the distributions of reproductive factors by family history, suggesting a lack of confounding by these variables. Analogous models were conducted for family history of breast or ovarian cancer. Each model generated an odds ratio (OR) and 95% confidence interval (CI). Tests for interaction between age and family history on TNBC status were conducted using likelihood ratio tests. All statistical analyses were performed using Stata 13.1 (StataCorp, College Station, TX).

## Results

A family history of breast cancer in a first-degree relative was reported by 13.1% of participants, and 24.1% reported breast cancer in a first- or second-degree relative (Table 1). The prevalence of a reported history of breast or ovarian cancer in a first-degree relative was 14.9%. Among women who were diagnosed at age <50 years, the prevalence of family history of breast cancer in a first- or second-degree relative increased to 27.4%. Few women reported more than one relative with breast or ovarian cancer.

**Table 1:** **Summary of family history of breast and/or ovarian cancer in the** ***Ella*** **study**

Family history characteristic	***n***/total (%)
Family history of any cancer in first- or second-degree relative	
No	510/1150 (44.4)
Yes	612/1150 (53.2)
Unknown due to adoption (participant does not know her blood relatives)	9/1150 (0.78)
Unknown (other reason)	19/1150 (1.65)
Family history of breast cancer	
In a first-degree relative	146/1112 (13.1)
In a first- or second-degree relative	264/1096 (24.1)
In a first- or second-degree relative and participant’s age of diagnosis < 50 y	138/504 (27.4)
Family history of breast or ovarian cancer	
In a first-degree relative	166/1112 (14.9)
In a first- or second-degree relative	293/1096 (26.7)
In a first- or second-degree relative and participant’s age of diagnosis < 50 y	147/504 (29.2)
Number of first-degree relatives with breast or ovarian cancer*	
0	946/1112 (85.1)
1	149/1112 (13.4)
2	15/1112 (1.35)
3	2/1112 (0.18)
Number of first- or second-degree relatives with breast or ovarian cancer	
0	803/1096 (73.3)
1	226/1096 (20.6)
2	45/1096 (4.11)
3+	22/1096 (2.01)

*No participants reported more than 3 first-degree relatives with breast or ovarian cancer.

Table 2 shows age and reproductive factors of women with at least one first-degree relative with breast cancer compared with women with no affected first-degree relatives. Analysis was restricted to the 914 women who had data for tumor subtype and knowledge of their family history of breast cancer in any relative. The mean age at diagnosis was similar between the two groups, as was parity, age at first full-term pregnancy, menstrual history, and breastfeeding history. However, differences by country of residence were shown for family history, wherein women recruited from Mexico were less likely to report a family history than women from the U.S.

**Table 2:** Risk factors in the Ella study by family history of breast cancer* ( ***n*** **=914)**

Risk factor	First-degree family history	No first-degree family history
	( ***n*** =135)	( ***n*** =779)
	***n*** (%)	***n*** (%)
Age at diagnosis, y		
< 50	58 (43.0)	361 (46.3)
≥ 50	77 (57.0)	418 (53.7)
Mean ± SD	53.6 ± 12.0	52.1 ± 12.4
Age at menarche, y		
<13	65 (48.1)	346 (44.4)
≥ 13	70 (51.9)	433 (55.6)
Mean ± SD	12.6 ± 1.6	12.8 ± 1.7
Parity		
Nulliparous	13 (9.6)	75 (9.6)
1–2 children	36 (26.7)	244 (31.3)
≥ 3 children	86 (63.7)	460 (59.1)
Mean ± SD	3.3 ± 2.2	3.1 ± 2.3
Age at first full term pregnancy, y		
< 21	41 (33.6)	288 (40.9)
21–24	38 (31.1)	183 (26.0)
≥ 25	43 (35.2)	232 (33.0)
Unknown	0	1 (.1)
Nulliparous	13	75
Mean ± SD	23.4 ± 5.6	22.8 ± 5.4
Time since last full-term pregnancy, y		
> 10	40 (69.0)	216 (60.7)
≤ 10	18 (31.0)	140 (39.3)
Postmenopausal or unknown	70	383
Premenopausal but nulliparous	7	40
Mean ± SD	12.8 ± 6.9	13.4 ± 8.0
Time from menarche to first pregnancy, y		
< 8	32 (26.2)	277 (39.3)
8–12	51 (41.8)	221 (31.5)
≥ 13	39 (32.0)	205 (29.1)
Unknown	0	1 (0.1)
Nulliparous	13	75
Mean ± SD	10.9 ± 5.6	9.9 ± 5.7
Lifetime duration of breastfeeding, mo		
Never	31 (25.4)	170 (24.1)
Up to 12	48 (39.3)	248 (35.2)
≥ 12	43 (35.2)	286 (40.6)
Nulliparous	13	75
Mean ± SD	15.6 ± 26.2	18.6 ± 31.5
Breastfeeding duration per birth, mo		
Never	31 (25.4)	170 (24.2)
> 0–5	54 (44.3)	269 (38.2)
≥ 5	37 (30.3)	265 (37.6)
Nulliparous	13	75
Mean ± SD	4.5 ± 6.6	5.3 ± 6.8
Menopausal status at diagnosis		
Pre	64 (47.8)	386 (50.2)
Post	70 (52.2)	383 (49.8)
Unknown	1	10
Age at menopause, y		
< 50	41 (58.6)	226 (59)
≥ 50	29 (41.4)	157 (41)
Premenopausal at diagnosis	64	386
Unknown	1	10
Mean ± SD	46.9 ± 6.9	47.3 ± 6.5
Hormone replacement therapy		
Never	117 (87.3)	674 (87.3)
Ever	17 (12.7)	98 (12.7)
Unknown	1	7
Hormone contraceptive use		
Never	58 (43.0)	345 (44.3)
Ever	77 (57.0)	434 (55.7)
Tertile of duration of menstruation, y		
7.6–27.35	21 (15.6)	172 (22.1)
27.4–33.45	40 (29.6)	201 (25.8)
33.5–50.5	65 (48.1)	343 (44)
Unknown	9 (6.7)	63 (8.1)
Mean ± SD	33.2 ± 6.7	32.1 ± 7.3
Country of residence		
Mexico	46 (11.5)	355 (88.5)
U.S.	89 (17.3)	424 (82.7)

*History of breast cancer in one or more first-degree relatives.

Among women with non-TNBC, 13.5% had a family history of breast cancer in a first-degree relative, compared with 21.6% of women with TNBC (Table 3). The prevalence of a family history of breast or ovarian cancer in a first-degree relative was 15.5% among patients with non-TNBC, compared with 24.3% among women with TNBC. After adjustment for age and country of residence, the OR (95% CI) for TNBC versus non-TNBC for women with a history of breast cancer in a first-degree relative was 1.98 (1.26–3.11), and that for women with an affected first- or second-degree relative was 2.04 (1.40–2.98). The odds of TNBC compared to non-TNBC were 1.93 (95% CI, 1.25–2.97) for women with a first-degree relative affected with breast or ovarian cancer.

**Table 3:** **Odds of TNBC compared to non-TNBC according to family history of breast and/or ovarian cancer (** ***n*** **= 914)**

Family history	Non-TNBC	TNBC	TNBC vs. non-TNBC	TNBC vs. non-TNBC
	***n*** (%)	***n*** (%)	OR (95% CI)*	OR (95% CI)**
Breast cancer in first-degree relatives				
No	663 (86.6)	116 (78.4)	1.00	1.00
Yes	102 (13.5)	32 (21.6)	1.78 (1.14–2.77)	1.98 (1.26–3.11)
Breast cancer in first- or second-degree relatives				
No	586 (76.5)	92 (62.2)	1.00	1.00
Yes	180 (23.5)	56 (37.8)	1.98 (1.37–2.87)	2.04 (1.40–2.98)
Breast or ovarian cancer in first-degree relatives				
No	647 (84.5)	112 (75.7)	1.00	1.00
Yes	119 (15.5)	36 (24.3)	1.75 (1.13–2.67)	1.93 (1.25–2.97)

*Crude odds ratio.

**Adjusted for age at diagnosis and country of residence (U.S. or Mexico).

We next conducted stratified analysis by age at diagnosis of the participant (Table 4). For all family history variables assessed, we observed a trend towards stronger associations with TNBC for younger (< 50 years) than older (≥ 50 years) women.

**Table 4:** **Odds of TNBC compared to non-TNBC according to family history of breast and/or ovarian cancer, stratified by participant’s age at diagnosis**

Family history	Age of diagnosis < 50 y		Age of diagnosis ≥ 50 y	
	TNBC	OR (95% CI)*	TNBC	OR (95% CI)*
	***n*** /total (%)		***n*** /total (%)	
Breast cancer in first-degree relatives				
No	66/361 (18.3)	1.00	50/418 (12.0)	1.00
Yes	21/58 (36.2)	2.61 (1.43–4.77)	11/77 (14.3)	1.38 (0.68–2.83)
	*P*-interaction = 0.14**			
Breast cancer in first- or second-degree relatives				
No	47/297 (15.8)	1.00	45/381 (11.8)	1.00
Yes	40/122 (32.8)	2.67 (1.63–4.37)	16/114 (14.0)	1.37 (0.73–2.56)
	*P*-interaction = 0.07**			
Breast or ovarian cancer in first-degree relatives				
No	66/357 (18.5)	1.00	46/402 (11.4)	1.00
Yes	21/62 (33.9)	2.31 (1.28–4.18)	15/93 (16.1)	1.61 (0.85–3.06)
	*P*-interaction = 0.36**			

*Adjusted for country of residence (U.S. or Mexico).

**Interaction between age at diagnosis and family history on TNBC subtype.

## Discussion

Our results support heterogeneity in risk of tumor subtype by family history, with TNBC having a stronger association with a positive family history than non-TNBC in this case series of women of Mexican descent. The magnitude of the associations was similar among women with breast cancer history in first-degree relatives, first- or second-degree relatives, as well as those with history of breast or ovarian cancer. There was a non-significant trend towards a stronger association between TNBC and positive family history among younger versus older women.

We conducted a review of the literature on prevalence of family history of breast cancer by breast tumor subtype and found 28 studies that have evaluated this relationship: 17 case–control (Rosato et al, 2013; Kawai et al, 2012; Bao et al, 2011; Yang et al, 2011; Dolle et al, 2009; Hines et al, 2008; Millikan et al, 2008; Yang et al, 2007; Rosenberg et al, 2006; Rusiecki et al, 2005; McCredie et al, 2003; Cotterchio et al, 2003; Britton et al, 2002; Huang et al, 2000; Yoo et al, 1997; Stanford et al, 1987; McTiernan et al, 1986), 9 cohort (Su et al, 2011; Phipps et al, 2011; Mavaddat et al, 2010; Setiawan et al, 2009; Welsh et al, 2009; Margolin et al, 2006; Colditz et al, 2004; Tutera et al, 1996; Potter et al, 1995), and 2 case-only studies (Song et al, 2013; Jiang et al, 2012) (Additional file 1: Table S1). Prevalence of family history among women with TNBC ranges from 5.3% in a Chinese case-case study (Song et al. 2013) to 22% among women with breast cancer age 40–84 years participating in the Breast Cancer Surveillance Consortium (Phipps et al. 2011) and 22.6% among younger women (age < 45 years) with breast cancer in the Seattle-Puget Sound area participating in a case–control study (Dolle et al. 2009). Of the 28 studies, only 9 assessed risk of TNBC compared to other subtypes by presence of family history: 3 studies found evidence of a stronger risk of TNBC compared to other subtypes (Yang et al. 2007, Su et al. 2011, Yang et al. 2011); 3 studies reported an elevated risk of TNBC as well as other subtypes (Millikan et al. 2008, Dolle et al. 2009, Phipps et al. 2011); 2 studies reported no significantly increased risk of TNBC (Welsh et al. 2009, Mavaddat et al. 2010); and one case-case study was descriptive and reported no significant differences for prevalence of family history by subtype (Song et al. 2013). In our review, we could not find any reports of family history and triple negative subtype in Hispanic/Latina women, making ours the first to report this association.

Compared to published reports on TNBC and family history of breast cancer, more data exist from studies that evaluated subtype based on only hormone receptor status with no information on HER2. Among the 26 case–control or cohort studies included in this literature review, 22 studies evaluated risk by hormone receptor status. Of these, 2 studies found a stronger association with ER+ versus ER– tumors (Welsh et al. 2009, Rosato et al. 2013). Four studies reported a stronger association for ER– versus ER+ tumors (Huang et al. 2000, Kawai et al. 2012), with one study finding this relationship only among Hispanic women (Hines et al. 2008) and another showing that the association held only among women with a first-degree relative diagnosed at age < 45 years (Tutera et al. 1996). The remaining 16 studies found no statistically significant differences in risk by hormone receptor status. Finally, the single published case-case study is from a Spanish population, which showed ER– or PR– cancers to be more likely than ER+ or PR+ to be associated with family history, but only among women age < 50 years (Jiang et al. 2012). It is important to note that of the 28 studies included in this review, only 2 included Hispanic/Latina women (Hines et al. 2008, Jiang et al. 2012), which underscores the tremendous scarcity of published data in this ethnic group.

Our findings are consistent with those of Hines et al. (2008), who reported a higher risk of ER– tumors associated with family history of breast cancer among Hispanic women in the southwestern U.S. Interestingly, they did not observe this association among non-Hispanic whites, a finding that further supports the potential role of *BRCA* founder mutations in Hispanics (Weitzel et al. 2007b). Weitzel et al. recently published results of a study of Hispanic/Latina women living in the southwestern U.S. who had either a personal or family history of breast and/or ovarian cancer; deleterious *BRCA* mutations were detected in 25% of participants (Weitzel et al. 2013). In a second report of breast cancer cases in Mexico, such mutations were detected in 28% of ovarian and 15% of breast cancer cases (Villarreal-Garza et al. 2014). These results suggest that *BRCA* mutations may account for a higher percentage of familial breast cancer in women of Mexican descent than in other racial/ethnic groups (Weitzel et al. 2013). Given that greater than 80% of *BRCA* mutated tumors are known to be TNBC (Penault-Llorca and Viale 2012), this provides a viable explanation for our results. Recent studies of genetic variation and breast cancer risk in Hispanic/Latina women have found further evidence of unique genetic factors that are important in this population (Fejerman et al. 2014). These findings further support the need to incorporate both ancestry and family history in studies of breast cancer risk factors.

It is recognized that accuracy in reporting family history of cancer is variable across studies, albeit reporting of breast cancer history has been shown to be of higher quality than that for other malignancies (Eerola et al. 2000, Murff et al. 2004, Mai et al. 2011). Family history reporting has also been found to be more accurate among first-degree relatives than second-degree or more-distant relatives (Eerola et al. 2000, Ivanovich et al. 2002, Mai et al. 2011). Data are sparse on accuracy of reporting of family history in different racial/ethnic or immigrant populations. Orom *et al*. noted that reporting is less accurate in immigrant groups and that non-white immigrants are less likely to report a family history of cancer than their white counterparts (Orom et al. 2008). John *et al*. showed that foreign-born Hispanic women reported a lower frequency of family history of breast cancer than U.S.-born Hispanics, which was evident among case and control women. Furthermore, there was a clear and significant increasing trend in prevalence of family history by level of acculturation in both pre- and post-menopausal women (John et al. 2005). Our results show that women in the U.S. had modestly higher self-reported family history of breast cancer than those in Mexico (17.3% versus 11.5%). This difference might reflect the underlying risk of breast cancer, awareness and knowledge across sub-groups, or both.

In spite of the potential for misreporting of family history, reporting is unlikely to be influenced by tumor subtype, which gives credibility to our findings. In addition, the fact that we observed consistently stronger, though non-significant, associations between family history and TNBC among younger women compared to older women may also be indicative of the high prevalence of *BRCA* mutation carriers in this population, who tend to be diagnosed with breast cancer at a younger age than do those with sporadic cancers (Weitzel et al. 2013). These findings underscore the importance of collecting a thorough family history in the oncology clinic and primary care setting, as it can affect approaches to prevention, treatment, and overall risk assessment (Murff et al. 2004, Orom et al. 2008, Wideroff et al. 2010, Mai et al. 2011). A recent American Society of Clinical Oncology consensus statement provides guidelines on the collection and use of cancer family history data (Lu et al. 2014). According to the guidelines, a minimum adequate family history for patients with cancer should include any family history in first- or second-degree relatives, with the type and age at diagnosis of each primary cancer. The history should be taken at the initial visit and updated periodically, with appropriate referrals made to genetic counseling for high-risk individuals.

Improvement in collection of family history through new tools and instruments targeting English- and Spanish-speaking Hispanic/Latina women in the U.S. should be a priority for future research, especially given that this group has been shown to be willing to participate in cancer genetic services (Ricker et al. 2006) and to embrace and act upon genetic counseling and risk assessment information (Lagos et al. 2008). Identification of a strong family history can ultimately affect treatment plans, screening practices, and prevention options both for patients and their relatives, which may include genetic testing for *BRCA* mutations.

Strengths of this study include the unique population comprising a large case series of women of Mexican descent residing in the U.S. and Mexico, as well as high participation rates, ranging between 95–99% (Martínez et al. 2010). Though our sample size was large, it was not sufficiently powered to allow for subset analyses based on age of diagnosis for both the participant and their affected first-degree relative. We had relatively few women reporting more than one affected relative and were thus unable to conduct analyses based on number of affected relatives. A further limitation of our study is that family history was self-reported and unable to be verified, which may have led to an underestimation of family history prevalence, as noted above. Lastly, because our study employed a clinic-based recruitment strategy and was not population-based, findings may not be broadly generalizable to the Hispanic/Latina community.

## Conclusions

In summary, results of the present study suggest a strong familial cancer association with TNBC, supporting etiologic heterogeneity by tumor subtype in this population of Hispanic/Latina women. This association may be related to the prevalence of *BRCA* founder mutations in this population, which are strongly associated with TNBC. Family history is an important tool to identify Hispanic/Latina women who may be at increased risk of TNBC, and could benefit from prevention and early detection strategies.

## Electronic supplementary material

Additional file 1: Table S1: Case-control, /cohort, and case-case studies evaluating family history by breast cancer subtype. (DOCX 53 KB)
