# Supplementary material for: Family history of breast and ovarian cancer and triple negative subtype in hispanic/latina women
Source: Springerplus. 2014 Dec 11;3:727. doi: 10.1186/2193-1801-3-727 (PMC4332916; doi:10.1186/2193-1801-3-727)
Supplement: Supplementary file 1 — Additional file 1: Table S1: Case-control, /cohort, and case-case studies evaluating family history by breast cancer subtype. (DOCX 53 KB) [file 40064_2014_1541_MOESM1_ESM.docx]

| **Supplementary Table 1. Case-control, /cohort, and case-case studies evaluating family history by breast cancer subtype** | | | | | | |
| --- | --- | --- | --- | --- | --- | --- |
| **Author, year** | **Study design** | **Population** | **Tumor Subtypes** | **Results** | | |
|  |  |  |  | **Prevalence** | | **Relative risk (cohort study) or odds ratio (case-control or case-case study) by subtype** |
| **Case-control/cohort studies** | | | | | | |
| Rosato, 2013  (Rosato, Bosetti et al. 2013) | Case-control | 1075 women with incident breast cancer, 1477 hospital controls | - ER+/PR+ - ER–/PR– - ER+ - ER– | Prevalence of family history in first-degree relative   - ER–: 16/243 (7%) - ER+: 82/832 (10%) - ER–/PR–: 11/182 (6%) - ER+/PR+: 68/716 (9%) | Family history in first-degree relative   - ER–: 1.4 (0.8–2.5) - ER+ : 2.2 (1.6–3.1) - ER–/PR–: 1.3 (0.7–2.6) - ER+/PR+: 2.2 (1.5–3.1)   Age of proband < 60 y   - ER–: 1.4 (0.7–2.7) - ER+: 1.7 (1.1–2.6) - ER–/PR–: 1.1 (0.5–2.6) - ER+/PR+: 1.6 (1.0–2.6)   Age of proband ≥ 60 y   - ER–: 1.5 (0.5–4.6) - ER+: 3.5 (2.0–6.0) - ER–/PR–: 1.9 (0.6–5.7) - ER+/PR+: 3.3 (1.9–5.9)   Age of youngest affected relative < 40 y   - ER–: 3.0 (0.9–10.0) - ER+: 1.8 (0.6–4.7) - ER–/PR–: 3.8 (1.1–13.6) - ER+/PR+: 2.1 (0.8–5.6)   Age of youngest affected relative ≥ 40 y   - ER–: 1.2 (0.6–2.3) - ER+: 2.2 (1.5–3.2) - ER–/PR–: 1.0 (0.5–2.3) - ER+/PR+: 2.1 (1.4–3.1) | |
| Kawai, 2012  (Kawai, Kakugawa et al. 2012) | Population-based case-control | 1092 women with breast cancer age ≥ 30 y and 3160 age-matched controls living in Japan | - ER+/PR+ - ER+/PR- - ER-/PR+ - ER–/PR– | Family history of breast cancer in first-degree relatives   - ER+/PR+: 48/572 (8%); - ER+/PR–: 17/133 (13%); - ER-/PR+: 3/24 (13%); - ER–/PR–: 33/271(12%) | Family history in first-degree relative vs. none   - ER+/PR+: 2.14 (1.49–3.08) - ER+/PR–: 3.52 (2.03–6.09) - ER–/PR+: 4.06 (1.15–14.31) - ER–/PR–: 3.51 (2.32–5.31)   (*P*-heterogeneity=0.044) | |
| Bao, 2011  (Bao, Shu et al. 2011) | Population-based case-control | Shanghai Breast Cancer Study: 2676 women with breast cancer age 20–-79 y and 3474 controls | - ER+/PR+ - ER+/PR– - ER–/PR+ - ER–/PR– | Percentage of women with breast cancer reporting positive family history in first-degree relative   - ER+/PR+: 4.4%; - ER-/PR: 4.63%; - ER+/PR-: 6.64%; - ER-/PR+: 5.12%; - Controls: 2.67%   (*P*=0.01 cases vs. controls,  *P*-heterogeneity=0.42 for comparisons between receptor status subtypes) |  | |
| Su, 2011  (Su, Zheng et al. 2011) | Population-based cohort | 2791 women with breast cancer age 20–75 y participating in the Shanghai Breast Cancer Survival Study | - Luminal A - Luminal B - HER2+ - TNBC | Percentage of women with positive family history   - Luminal A: 4.5% - Luminal B: 4.5% - HER2+: 4.45% - TNBC: 7.78%   (*P*-heterogeneity=0.048) |  | |
| Phipps, 2011  (Phipps, Buist et al. 2011) | Prospective cohort | 1,054,466 women age 40–84 y participating in the Breast Cancer Surveillance Consortium; 11,039 women with breast cancer and subtype information | - ER+; - ER–/PR–/HER2+ - TNBC | Overall prevalence of family history in first-degree relative   - 15%   By subtype   - ER+: 22% - ER–/PR–/HER2+: 20% - TNBC: 22%   Overall prevalence among cases   - Age < 55 y: 13% - Age ≥ 55 y: 16% | Positive family history in ≥ 1 first-degree relative vs. none   - TNBC: 1.73 (1.43–2.09) - ER+: 1.62 (1.54–1.70); - ER–/PR–/HER2+: 1.56 (1.15–2.13)   At least two affected first-degree relatives vs. none   - TNBC: 2.66 (1.66–4.27) - ER+: 2.05 (1.79–2.36) - ER–/PR–/HER2+: 2.25 (0.99–5.08) | |
| Yang, 2011  (Yang, Chang-Claude et al. 2011) | Pooled case-case of 12 population-based studies and case-control comparison | 35,568 breast cancer patients from 34 studies, age range 18–100 y, participating in Breast Cancer Association Consortium | - ER+ - PR+ - ER+/PR+ - ER+/HER2+ - HER2+ - PR+/HER2+ - ER+/PR– - ER–/PR+ - ER–/PR– - TNBC | Prevalence of family history of breast cancer in a first-degree relative   - All cases: 20% - ER+: 21% - ER–: 19% - ER+/PR+: 22% - ER–/PR–: 19% - ER+ or PR+/HER2–: 19% - ER+ or PR+/HER2+: 16% - ER–/PR–/HER2+: 16% - TNBC: 18% | Case-case comparisons of positive vs. negative family history of breast cancer in a first-degree relative   - ER– vs. ER+: 0.95 (0.89–1.02) - PR– vs. PR+: 0.95 (0.89–1.01) - TNBC vs. ER+ or PR+/HER2–: 0.99 (0.86–1.14) - TNBC with core basal phenotype marker (CK5/6, CK5, EGFR) vs. ER+ or PR+/HER2–: 1.38 (1.08–1.75) | |
| Mavaddat, 2010  (Mavaddat, Pharoah et al. 2010) | Retrospective cohort | 4590 index cases—women with breast cancer, < age 55 y, diagnosed since 1991; 1163 incident cases among 14,439 first-degree relatives diagnosed between 1996–2010 and age < 70 y at diagnosis | - ER+/PR+ - ER+/PR– - ER–/PR+ - ER+ or PR+/HER2+ - ER–/PR–/HER2+ - TNBC | Prevalence of family history in first-degree relative among   - All cases: 15.9% - ER+ or PR+: 16.3% - Luminal HER2+: 16.7% - TNBC: 13.7% | Index case age < 50 y at diagnosis and first-degree relative < 50   - ER–: 3.40 (2.04–4.75) - ER+: 2.70 (2.01–3.39)   Index case < 50 y at diagnosis and first-degree relative age 50–84 y   - ER–: 1.42 (0.81–2.03)   Index age > 50 y at diagnosis and first-degree relative age < 50 y   - ER–: 2.56 (1.31–3.81) - ER+: 1.72 (1.33–2.11)   Index case > 50 y at diagnosis and first-degree relative age 50–84 y:   - ER–: 1.40 (1.01–1.80) - ER+: 1.78 (1.59–1.98)   Index case any age, first-degree relative age < 50 y,   - ER–: 2.96 (2.04–3.87) - ER+: 2.05 (1.70–2.40)   Index case any age, first-degree relative age 50–84 y,   - ER–: 1.41 (1.08–1.74) - ER+: 1.76 (1.59–1.93)   Case-case analysis for family history in first-degree relative   - ER+ vs. ER-: 1.04 (0.85–1.28) - HER2+ vs. HER2–: 1.15 (0.79–1.68) | |
| Setiawan, 2009  (Setiawan, Monroe et al. 2009) | Prospective cohort | 84,427 women in the Multiethnic Cohort Study age 45–75 y; 2543 breast pre-cancer cases | - ER+/PR+ - ER+/PR– - ER–/PR+ - ER–/PR– | Prevalence of family history in first-degree relative   - ER+/PR+: 17.6% - ER+/PR–: 18%; - ER–/PR–: 18.8% | Family history of breast cancer in first-degree relative vs. none   - ER+/PR+: 1.63 (1.43–1.80) - ER+/PR–: 1.72 (1.27–2.34) - ER–/PR–: 1.91 (1.51–2.43) | |
| Dolle, 2009  (Dolle, Daling et al. 2009) | Case-control | 897 women with breast cancer age 20–45 y and 1569 age-matched controls | - TNBC - Non-TNBC | Prevalence of breast cancer in first-degree relative   - All cases: 20.8% - TNBC: 22.6% - Non-TNBC: 20.3% | Family history of breast cancer in first-degree relative   - TNBC: 3.5 (2.1–5.9) - Non-TNBC: 2.8 (2.0–4.0)   (*P*-heterogeneity=0.7)  Family history of breast cancer in second-degree relative   - TNBC: 1.8 (1.2–2.8) - Non-TNBC: 1.7 (1.3–2.2) | |
| Welsh, 2009  (Welsh, Buist et al. 2009) | Cohort | 75,169 women in Western Washington cohort, 1087 diagnosed with breast cancer, age ≥ 40 y | - ER+/PR+ - ER+/PR- - ER-/PR- - Luminal A - Luminal B - HER2+ - Basal |  | Any first-degree relative vs. none   - ER+/PR+: 1.49 (1.24–1.87) - ER+.PR–: 2.43 (1.47–4.01) - ER–/PR–: 1.36 (0.91–2.03) - Luminal A: 1.78 (1.42–2.23) - Luminal B: 2.10 (0.84–5.22) - HER2+: 2.91 (0.84–10.1) - Basal: 1.01 (0.53–1.90)   Second-degree relative only vs. none   - ER+/PR+: 1.11 (0.91–1.37) - ER+.PR–: 1.60 (0.89–2.87) - ER–/PR–: 1.02 (0.66–1.59) - Luminal A: 1.23 (0.94–1.61) - Luminal B: 1.98 (0.79–4.94) - HER2+: 4.87(1.62–14.6) - Basal: 0.33 (0.12–0.93) | |
| Hines, 2008  (Hines, Risendal et al. 2008) | Case-control | 1537 women with breast cancer age 25–79 y and 2452 age- and ethnicity-matched controls participating in 4-Corners Breast Cancer Study | - ER– - ER+ | Prevalence of family history of breast cancer in first-degree relative,  non-Hispanic white:   - ER–: 18% - ER+: 23%   Hispanic:   - ER–: 22% - ER+: 14% | Hispanic women with positive family history vs. none   - ER–: 2.44 (1.51–3.95) - ER+: 1.08 (0.74-1.57)   Non-Hispanic white women with positive family history vs. none   - ER–; 1.43 (0.95–2.16) - ER+: 1.80 (1.44-2.25) | |
| Millikan, 2008  (Millikan, Newman et al. 2008) | Case-control and case-case | Carolina Breast Cancer study: 1803 cases and 1564 matched controls;  age 20-74 y | - Luminal A - Luminal B - HER2+ - Basal - Unclassified - TNBC (included basal and unclassified) | Prevalence of family history   - Luminal A: 16.9% - Basal-like: 16.2% - HER2+: 15.2% - Luminal B: 18.4% - Unclassified: 16.8% | Family history vs. none, case-control   - Luminal A: 1.5 (1.2–1.9) - Basal-like: 1.7 (1.1–2.5)   Family history vs. none, case-case with referent Luminal A   - Basal: 1.0 (0.7–1.5) - HER2+: 0.9 (0.5–1.6) - Luminal B: 1.1 (0.7–1.8) - Unclassified: 1.0 (0.6–1.7) | |
| Yang, 2007  (Yang, Sherman et al. 2007) | Case-control | 804 breast cancer patients and 2502 population-matched controls participating in a Polish Breast Cancer Study;  age 20–74 y | - Luminal A - Luminal B - HER2+ - Basal - Unclassified | Prevalence of family history in first-degree relative   - Luminal A: 10% - Luminal B: 10% - HER2: 13% - Basal like: 16% - Unclassified: 6% | Family history in first-degree relatives vs. no family history   - Luminal A: 1.72 (1.21–2.45) - Luminal B: 2.31 (0.88–6.08) - HER2+: 2.35 (1.03–5.38) - TNBC: 3.17 (1.69–5.92)   (*P*-heterogeneity=0.11 for TNBC vs. Luminal A) | |
| Rosenberg, 2006  (Rosenberg, Einarsdottir et al. 2006) | Case-control | 1165 post-menopausal Swedish women age 50–74 y diagnosed with breast cancer with available ER/PR status; 3065 age-matched controls | - ER–/PR– - ER+/PR– - ER–/PR+ - ER+/PR+ | Prevalence of family history in first-degree relative   - ER–/PR–: 14% - ER+/PR–: 14% - ER–/PR+: 22% - ER+/PR+: 17% | Family history in first-degree relative vs. none   - ER–/PR–: 1.7 (1.2–2.3) - ER+/PR–: 1.7 (1.2–2.4) - ER–/PR+: 2.7 (1.5–4.9) - ER+/PR+: 2.0 (1.7–2.5)   (*P*-heterogeneity=0.33) | |
| Margolin, 2006  (Margolin, Johansson et al. 2006) | Cohort | 489 women newly diagnosed with breast cancer in Sweden | - ER+ - ER– | Overall prevalence of family history   - 35%   Women with ≥ 2 first- or second-degree relatives with breast cancer   - ER–: 10% - ER+: 72%   Women without family history of breast cancer (sporadic)   - ER–: 15%; - ER+: 81%   (*P*-heterogeneity=0.17)  Women diagnosed with breast cancer age ≤ 50 y and with a first- or second-degree relative with breast cancer   - ER–: 28% - ER+: 70%   Without family history of breast cancer:   - ER–: 18% - ER+: 80%   (*P*-heterogeneity=0.27) |  | |
| Rusiecki, 2005  (Rusiecki, Holford et al. 2005) | Case-control | 420 women with breast cancer age 40–80 y, 406 controls | - ER+/PR+ - ER–/PR– - ER+/PR– - ER–/PR+ | Prevalence of family history, all cases   - 24% | Family history of breast cancer in a first-degree relative vs. none   - ER+/PR+: 1.7 (1.0–2.8) - ER–/PR–: 1.1 (0.7–19) - ER+/PR–: 0.8 (0.4–1.5) - ER–/PR+: 1.6 (0.8–3.4)   (No statistically significant differences observed) | |
| Colditz, 2004  (Colditz, Rosner et al. 2004) | Cohort | Nurses’ Health Study: 66,145 women age 30–55 y at time of enrollment with 2096 incident cases of breast cancer | - ER+/PR+ - ER+/PR– - ER–/PR+ - ER–/PR– |  | Family history of breast cancer in a first-degree relative vs. none   - ER+/PR+: 1.45 (1.25–1.68) - ER+/PR–: 1.79 (1.36–2.34) - ER–/PR+: 1.80 (1.03–3.17) - ER–/PR–: 1.70 (1.32–2.19) | |
| McCredie, 2003  (McCredie, Dite et al. 2003) | Case-control | 765 cases and 564 controls in population-based Australian Breast Cancer Family Study, age < 40 y | - ER+/PR+ - ER+/PR– - ER–/PR+ - ER–/PR– | Prevalence of first-degree relative with breast cancer   - ER+/PR+: 9% - ER+/PR–: 12% - ER–/PR+: 8% - ER–/PR–: 11% | Family history of breast cancer in first-degree relative vs. none   - ER+/PR+: 2.5 (1.4–4.6) - ER+/PR-: 3.4 (1.1–11) - ER–/PR+: 1.8 (0.6–4.8) - ER–/PR–: 3.4 (1.1–6.4) | |
| Cotterchio, 2003  (Cotterchio, Kreiger et al. 2003) | Case-control | Two population-based case-control studies with 3278 women with breast cancer age 25–74 y, for whom hormone receptor status could be obtained, and 3691 age-matched controls | - ER+/PR+ - ER–/PR– |  | Family history of breast cancer, pre-menopausal cases   - ER+/PR+: 1.73 (1.08–2.76) - ER–/PR–: 2.35 (1.35–4.10)   (*P*-heterogeneity=0.32) | |
| Britton, 2002  (Britton, Gammon et al. 2002) | Case-control | 1556 women with breast cancer age 20–44 y, 1397 age-matched controls | - ER+/PR+ - ER+/PR– - ER–/PR+ - ER-/PR- |  | Family history of breast cancer in first-degree relative vs. none   - ER+/PR+: 2.31 (1.67–3.18) - ER+/PR–: 1.69 (0.89–3.22) - ER–/PR+: 1.93 (1.03–3.61) - ER–/PR–: 2.53 (1.74–3.69) | |
| Huang, 2000  (Huang, Newman et al. 2000) | Case-control | 862 women with breast cancer from the Carolina Breast Cancer study age 20–75 y, 790 age-matched controls | - ER+/PR+ - ER+/PR– - ER–/PR+ - ER–/PR– |  | First-degree family history of breast or ovarian cancer vs. none   - ER+/PR+: 1.2 (0.8–1.7) - ER+/PR–: 1.5 (0.8–3.0) - ER–/PR+: 1.6 (0.7–3.2) - ER–/PR–: 1.8 (1.2–2.7)   (No statistically significant differences observed) | |
| Yoo, 1997  (Yoo, Tajima et al. 1997) | Case-control | 458 women with breast cancer for whom ER status was known, age ≥ 25 y, and 21,714 cancer-free controls in Japan | - ER+ - ER– - PR+ - PR– - ER+/PR+ - ER+/PR– - ER–/PR+ - ER–/PR– |  | Family history in a first-degree relative vs. none   - ER+: 1.53 (0.87–2.69) - ER–: 1.06 (0.88–1.28) - PR+: 1.88 (1.01–3.47) - PR–: 1.36 (0.61–1.01) - ER+/PR+: 1.73 (0.88–3.40) - ER+/PR–: 1.22 (0.45–3.32) - ER–/PR+: 3.08 (0.72–13.2) - ER–/PR–: 1.48 (0.65–3.37)   (No statistically significant differences observed) | |
| Tutera, 1996  (Tutera, Sellers et al. 1996) | Cohort | Iowa Women’s Health Study: 41,837 postmenopausal women, 610 diagnosed with breast cancer who had known ER/PR status | - ER+/PR+ - ER+/PR– - ER–/PR+ - ER–/PR– | Prevalence of family history in a first-degree relative   - All cases: 16% - ER+/PR+: 14.9% - ER+/PR–: 8.4% - ER–/PR+: 23.5% - ER–/PR–: 17.9% | Family history in mother   - ER+PR+: 1.28 (0.89–1.86) - ER+/PR–: 0.52 (0.16–1.64) - ER–/PR+: 2.17 (0.50–9.47) - ER–/PR–: 1.11 (0.45–2.73)   Family history in sister   - ER+/PR+: 1.22 (0.85–1.76) - ER+/PR–: 0.80 (0.33–1.98) - ER–/PR+: 1.99 (0.45–8.75) - ER–/PR–: 1.91 (0.95–3.83)   Onset of cancer in a first-degree relative at age ≤ 45 y   - ER+/PR+: 0.90 (0.49–1.64) - ER+/PR–: 0.69 (0.17–2.82) - ER–/PR+: 2.28 (0.30–17.53) - ER–/PR–: 3.33 (1.52–7.29)   Onset of cancer in a first-degree relative at age > 45 y   - ER+/PR+: 1.28 (0.95–1.72) - ER+/PR–: 0.91 (0.45–1.82) - ER–/PR+: 3.21 (0.99–10.42) - ER–/PR–: 1.18 (0.58–2.38) | |
| Potter, 1995  (Potter, Cerhan et al. 1995) | Cohort | Iowa Women’s Health Study: 37,105 women age 55–69 y, 610 cases of invasive breast cancer with known hormone receptor status | - ER+/PR+ - ER+/PR– - ER–/PR+ - ER–/PR– | Prevalence of family history by subtype   - ER+/PR+: 14.9% - ER+/PR–: 8% - ER–/PR+: 23.5% - ER–/PR–: 17.9 | Family history of breast cancer in a first-degree relative vs. none   - ER+/PR+: 1.25 (0.95–1.65) - ER+/PR–: 0.66 (0.32–1.36) - ER–/PR+: 2.24 (0.73–6.87) - ER–/PR–: 1.57 (0.88–2.80) | |
| Stanford, 1987  (Stanford, Szklo et al. 1987) | Case-control | 458 women with breast cancer and known ER status, age 20–54 y, and 568 age-matched controls | - ER+ - ER– |  | Family history of breast cancer in a first-degree relative   - ER+: 2.52 (1.3–4.7) - ER–: 2.18 (1.2–4.0)   Family history of breast cancer in a in second-degree relative   - ER+: 1.56 (1.0–2.4) - ER–: 1.34 (0.9–2.0) | |
| McTiernan, 1986  (McTiernan, Thomas et al. 1986) | Case-control | 240 women with breast cancer, age 25–54 y, with known ER status, and 332 age-matched controls | - ER+ - ER– | Prevalence of family history in a first-degree relative   - ER+: 9.8% - ER–: 13.4% | Family history of breast cancer in a first degree relative vs. none   - ER+: 1.5 (0.70-3.0) - ER-: 1.9 (0.78-4.2)   First- or second-degree relative   - ER+: 1.7 (0.38–7.9) - ER–: 4.7 (0.78–28) | |
| **Case-only studies** | | | | | | |
| Song 2013  (Song, Huang et al. 2013) | Case-case | 4211 women with breast cancer in China, age 21–86 y | - ER+/– - PR+/– - ER+/PR+ - ER–/PR– - HER2+/– - Luminal A - Luminal B - ER–/PR–/HER2+ - TNBC | Prevalence of family history in a first-degree relative   - ER+: 3.5% - ER–: 4.1% - PR+: 3.2% - PR–: 4.5% - HER2+: 2.4% - HER2–: 4.4% - ER+/PR+: 3.1% - ER+/PR–: 5.4% - ER–/PR+: 3.6% - ER–/PR–: 4.2% - Luminal A: 4.0% - Luminal B: 2.1% - ER–/PR–/HER2+: 2.9% - TNBC: 5.3%   (*P*-heterogeneity=0.52) |  | |
| Jiang, 2012  (Jiang, Castelao et al. 2012) | Case-case | 645 women diagnosed with operable invasive breast cancer, age 25–85 y | - ER+ or PR+ - ER–/PR– | Prevalence of family history in a first-degree relative   - All: 125/675 (19%) - ER+/PR+: 91/504 (18%) - ER–/PR–: 34/141 (24%)   Prevalence of family history among women age < 50 y   - ER+/PR+: 40/138 (29%) - ER–/PR–: 21/40 (53%)   Prevalence of family history among women age ≥ 50 y   - ER+/PR+: 51/366 (14%) - ER–/PR–: 13/101 (13%) | Family history vs. none, adjusted for age at diagnosis   - ER–/PR– vs. ER+ or PR+: 1.43 (0.91–2.26)   Age < 50 y, positive family history   - ER–/PR– vs. ER+ or PR+: 2.79 (1.34–5.81) - ER– vs. ER+: 2.66 (1.32–5.39) - PR+ vs. PR–: 1.40 (0.72–2.72)   Age > 50 y, positive family history   - ER–/PR– vs. ER+ or PR+: 0.91 (0.48–1.76) | |
